# DR_SEQAN: a PC/Windows-based software to evaluate drug resistance using human immunodeficiency virus type 1 genotypes

**DOI:** 10.1186/1471-2334-6-44

**Published:** 2006-03-08

**Authors:** César Garriga, Luis Menéndez-Arias

**Affiliations:** 1 Centro de Biología Molecular "Severo Ochoa", Consejo Superior de Investigaciones Científicas – Universidad Autónoma de Madrid, Cantoblanco, 28049 Madrid, Spain

## Abstract

**Background:**

Genotypic assays based on DNA sequencing of part or the whole reverse transcriptase (RT)- and protease (PR)-coding regions of the human immunodeficiency virus type 1 (HIV-1) genome have become part of the routine clinical management of HIV-infected individuals. However, the results are difficult to interpret due to complex interactions between mutations found in viral genes.

**Results:**

DR_SEQAN is a tool to analyze RT and PR sequences. The program output includes a list containing all of the amino acid changes found in the query sequence in comparison with the sequence of a wild-type HIV-1 strain. Translation of codons containing nucleotide mixtures can result in potential ambiguities or heterogeneities in the amino acid sequence. The program identifies all possible combinations of 2 or 3 amino acids that derive from translation of triplets containing nucleotide mixtures. In addition, when ambiguities affect codons relevant for drug resistance, DR_SEQAN allows the user to select the appropriate mutation to be considered by the program's drug resistance interpretation algorithm. Resistance is predicted using a rule-based algorithm, whose efficiency and accuracy has been tested with a large set of drug susceptibility data. Drug resistance predictions given by DR_SEQAN were consistent with phenotypic data and coherent with predictions provided by other publicly available algorithms. In addition, the program output provides two tables showing published drug susceptibility data and references for mutations and combinations of mutations found in the analyzed sequence. These data are retrieved from an integrated relational database, implemented in Microsoft Access, which includes two sets of non-redundant core tables (one for combinations of mutations in the PR and the other for combinations in the RT).

**Conclusion:**

DR_SEQAN is an easy to use off-line application that provides expert advice on HIV genotypic resistance interpretation. It is coded in Visual Basic for use in PC/Windows-based platforms. The program is freely available under the General Public License. The program (including the integrated database), documentation and a sample sequence can be downloaded from

## Background

According to recent reports of the World Health Organization, there are more than 40 million people infected with the human immunodeficiency virus (HIV) around the world [1]. Sixty-five percent of them live in sub-Saharan Africa. In Western Europe, an estimated 720,000 people are living with HIV, and in the United States this figure goes up to 1,200,000. Since the introduction in the mid-90s of highly active antiretroviral therapies, mortality due to HIV infection has decreased in those countries where the population has access to antiretroviral treatments. However, even in developed countries the number of infected people is increasing.

At present, there are around 20 compounds approved for clinical use against HIV. These drugs are usually either inhibitors of the viral reverse transcriptase (RT), or inhibitors of the viral protease (PR), although a novel peptide (known as enfuvirtide) which blocks viral entry by interfering with the process involving fusion of the viral envelope and the host cell membrane has been recently licensed [2]. Despite the introduction of potent antiretroviral therapies, drug resistance is still a major hurdle to achieve complete suppression of viral replication in many patients, and compromises the long-term efficacy of antiretroviral regimens.

Since the introduction of antiretroviral therapy with RT and/or PR inhibitors, evidence of drug resistance has been observed and mutations conferring drug resistance have been identified [3,4]. Since the number of approved antiretroviral drugs has been steadily increasing, the need for optimizing the treatment based on genotypic information is becoming more evident. Antiretroviral drug resistance testing has become part of the routine clinical management of HIV-infected individuals in developed countries. Genotypic assays based on DNA sequencing of the HIV-1 *pol *gene (including part of the RT-coding region and the whole PR) are frequently done to help physicians choose antiretroviral drugs by identifying drug resistance mutations in the plasma virus of infected patients. These assays have a faster turnaround time, are easier to perform and are less expensive than phenotypic assays (for reviews, see [5,6]). Nevertheless, a major difficulty with resistance genotyping is the interpretation of results.

A large number of genotypic resistance interpretation tools have been developed in recent years and are available by different means [7,8]. These include lists of mutations relevant for drug resistance, rule-based algorithms, and mathematical models based on correlations found in databases containing genotypes and drug susceptibility data [4,9-15]. Although approaches based on mathematical models such as the support vector machine algorithm [11] or the linear regression model [10] provide objective quantitative assessments of drug resistance, rule-based algorithms are more efficient when estimating the effects of mutations underrepresented in the genotype-phenotype data sets used by those programs. In addition, sometimes there is a lack of consistency between clinical response and phenotypic data that can be corrected only by specific rules. Despite the large number of studies focusing on the role of mutations in drug resistance, there are many questions unanswered on the contribution of amino acid substitutions in the viral resistance phenotype and its clinical implications.

In this scenario, DR_SEQAN (Drug Resistance SEQuence ANalyzer) has been developed to identify those mutations in the PR- and RT-coding regions of the HIV-1 genome that lead to amino acid substitutions (*i.e*. non-synonymous mutations), which are subsequently classified as relevant or non-relevant for drug resistance. An interpretation algorithm is used to predict viral drug susceptibility. In addition, the program provides a report with published phenotypic data for mutations or combinations of mutations found in the query sequence.

## Implementation

The source code of DR_SEQAN has been written in Visual Basic 6.0 (Microsoft) (source code available on request), and developed for a PC/Windows environment.

### Sequence data

HIV drug resistance tests involve the determination of the viral genotype. For this purpose, viral RNA is extracted from plasma and the RT- and PR-coding regions are amplified by using a nested reverse-transcription polymerase chain reaction (RT-PCR). RT-PCR products are then subjected to automatic sequencing. Data input for DR_SEQAN is the obtained proviral DNA sequence. Sequence data can be introduced either as a text file (without headings or in FASTA format), or as a list of mutations. The query sequence can be uploaded from a file (*i.e*. FILE.TXT), or pasted in the appropriate window.

When the program is executed, the coding nucleotide sequence is automatically translated. The obtained amino acid sequences are then compared with the PR and RT sequences of the reference wild-type strain HIV-1_HXB2 _[GenBank:K03455], using BLAST [16]. The obtained alignment is used to identify amino acid differences between the query and reference sequences. For the program to run, the analyzed sequence must be at least 260 nucleotides long. Although DR_SEQAN accepts partial nucleotide sequences, they should contain one of the conserved motifs ('DTGAD' or 'GPTP') of the PR [17] to allow PR sequence analysis.

It should be noted that conventional population-based sequence approaches allow the characterization of the dominant viral species, but do not reliably detect variants that comprise less than 20 % of the total virus population. However, the presence of resistant minorities can lead to sequence heterogeneities that often result in unidentifiable residues. The program accepts mixtures of 2, 3 or 4 nucleotides in any position of the query sequence when the appropriate single-letter codes are used: B for (for the mixture of C, G and T), D (for A + G + T), H (for A + C + T), K (for G + T), M (for A + C), N (for A + C + G + T), R (for A + G), S (for C + G), V (for A + C + G), W (for A + T), and Y (for C + T). DR_SEQAN keeps to a minimum the number of unidentified residues (typically represented with an 'X'), by assuming all cases where mixtures of 2, 3 or 4 nucleotides do not result in an ambiguous amino acid sequence determination (*i.e*. the triplet GGN is translated as G). In addition, it provides information on any potential combination of 2 or 3 amino acids that could be derived from codons containing nucleotide mixtures (*i.e*. TTM is translated as [F/L], BTC as [F/L/V], etc.).

### Prediction algorithm

Sequence alignments allow the identification of amino acid differences between the query sequence and the sequence of the wild-type HIV-1 clone used as a reference. Then, DR_SEQAN makes a drug resistance prediction using a rule-based algorithm based on current evidence and authors' expertise. The rules used for the genotypic interpretation are based on correlations between the effects of mutations and/or combinations of mutations in drug resistance as observed *in vitro *or in the clinical setting [15]. The rules used by DR_SEQAN version 1.0 are given as supplementary information [see Additional file 1], but are also accessible through the help command of the program.

It should be noted that only certain residues could be relevant for resistance when nucleotide mixtures appear in the sequence. For example, at position 215 of the RT-coding region, the substitutions T215F and T215Y contribute to zidovudine resistance, but T215D or T215S have no impact on drug susceptibility [3,4]. In this case, the significance of T215X could be uncertain. However, when this uncertainty occurs at codons related to drug resistance, the program displays a window where the sequence ambiguities (derived amino acid mixtures) are shown together with the actual codon found in the sequence. A series of buttons allow the user to select for the wild-type or the appropriate mutation to be considered by the prediction algorithms. If no selection is made, the prediction algorithm assumes that the mutation found at the corresponding position would be the one having the largest effect on drug resistance.

### Phenotypic data

The impact on phenotypic resistance of mutations found in the query sequence is retrieved from an integrated relational database (implemented in Microsoft Access), which includes 2 sets of non-redundant core tables (one for the PR and the other one for the RT). These tables contain information on phenotypic drug susceptibility (fold-increase of the IC_50 _relative to the wild-type reference virus) as well as references for each drug and combination of amino acid substitutions. The database contains 6,835 entries, with drug susceptibility data for all 19 licensed compounds, collected from 263 published papers [15].

## Results and discussion

The interpretation of genotypic drug resistance tests is difficult due to the complex mutational patterns that appear in HIV isolates from infected patients, particularly those that have been heavily-treated. Mutational pattern complexity increases as more new drugs become part of the antiretroviral treatments. Available genotypic interpretation algorithms range from sophisticated machine learning-based methods to look-up tables [7,8]. However, algorithms need to be updated frequently, and any additional information on the effects of specific mutations on drug resistance should be helpful to make a decision on treatment.

**Figure 1:**
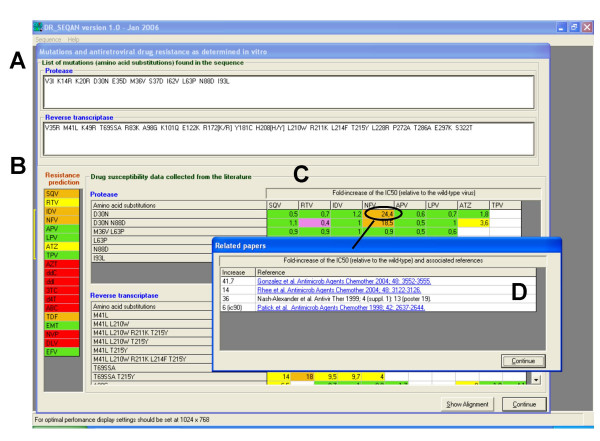
**DR_SEQAN output window**. (A) List of mutations found in the PR- and RT-coding regions. (B) Predicted resistance as determined with the program's algorithm (red, high-level; orange, significant level; yellow, partial resistance; green, drug-sensitive). (C) Drug susceptibility data reported in the literature for different combinations of mutations found in the query sequence. (D) References and IC_50 _values reported for the indicated inhibitor and combination of mutations (this window appears by double clicking on the corresponding cell).

The output generated by DR_SEQAN (Figure 1) consists of three parts: (i) a list of amino acid substitutions in the PR- and RT-coding regions, indicating those changes related to drug resistance; (ii) a prediction of the expected level of resistance to all available antiretroviral drugs, as determined using a rule-based algorithm; and (iii), a chart with published drug susceptibility data for each mutation or combination of mutations found in the query sequence, where the relative increases of the IC_50_s for each inhibitor are given.

The program makes a drug resistance prediction using a rule-based algorithm [see Additional file 1]. Results, representing high-level, significant or partial resistance to each inhibitor are indicated in red, orange or yellow, respectively (Figure 1). Green is used to indicate that the virus is expected to remain susceptible to the drug. When either the PR or the RT sequence is missing, resistance to the corresponding inhibitors cannot be predicted, and the lack of information is represented in white.

DR_SEQAN's interpretation algorithm is an updated version of an earlier set of rules that showed good performance when tested on clinical samples [18]. Now, we have compared the predictions given by DR_SEQAN with those obtained using four HIV-1 genotypic resistance interpretation algorithms, available from Virology Networks (Retrogram version 1.6) [19], the French Agency for AIDS Research (ANRS algorithm, version 9/04) [20,21], the Stanford University HIV Drug Resistance Database (HIVDB version 3/05) [11,22], and the Rega Institute (RegaInst version 6.2) [9]. These algorithms use a three- or four-level category classification of predicted resistance, with interpretations such as 'high-level resistance', 'intermediate resistant', 'possible resistance', or 'recommended drug to be used for treatment', among others. Equivalencies between the recommendations given by the algorithms are shown in Table 1.

**Table 1:** Consensus categories and their significance according to the algorithms used in the comparison.

	Algorithms				
	
Category	DR_SEQAN	Retrogram	ANRS	HIVDB	RegaInst
S (green)	Virus expected to be susceptible to the drug	The corresponding drug can be used (susceptible)	Susceptible	Potential low-level resistance (or susceptible)	Susceptible
I (yellow)	Partial/low-level resistance	Consider the use of this drug if no 'S' class drugs are available	Possible resistance	Intermediate/low-level resistance	Intermediate resistant
IR (orange)	Significant resistance predicted	Consider the use of this drug if no 'S' or 'I' class drugs are available			
R (red)	Predicted resistance levels are very high	To be used only if 'S', 'I' or 'IR' classes are not available	Resistance	High-level resistance	Resistant

There are some relevant differences between the algorithm of DR_SEQAN and the others. First, DR_SEQAN provides a drug resistance interpretation for each of the 19 RT and PR inhibitors licensed for treatment of HIV infection, while providing updated phenotypic data collected from the literature. Second, there are differences in DR_SEQAN's algorithm that affect amino acid substitutions that are not considered by the others. For example, Retrogram does not take into account several mutations that could be relevant for drug resistance, such as R8Q, K20T, L33F, E34Q, M36L, G48M, I54A, I54S, Q58E, A71I, and G73A/C/S in the PR-coding region. Third, DR_SEQAN warns on the presence and number of antagonistic mutations, assuming their consequences for zidovudine (AZT), tenofovir, delavirdine and amprenavir resistance. The rules used by other algorithms (*i.e*. Retrogram, ANRS or RegaInst) do not take into account the effects of antagonistic mutations, or make a limited used of them (*i.e*. HIVDB assumes only the suppressive effects of K65R, L74V, L100I, Y181C and M184I/V on AZT resistance, and M184V on tenofovir resistance mediated by K65R). However, DR_SEQAN assumes the effects of mutations such as W88G, E89K or E89G that confer resistance to foscarnet while increasing AZT susceptibility, or K20T whose antagonist effect on amprenavir resistance has been demonstrated [14,15]. Another relevant feature of DR_SEQAN is that its output shows those positions involved in drug resistance, whose sequence has not been provided.

The prediction accuracy of the algorithms was tested using the Genotype-Phenotype Dataset, version 2.0 (release on Nov 1, 2004), available from the Stanford database [21]. This data set contains drug susceptibility data obtained with the PhenoSense™ assay, available from Monogram Biosciences Inc. (formerly Virologic) [22]. After excluding the data of those RTs whose mutation lists were not complete, and the data of PR sequences having poorly defined amino acid substitutions (*i.e*. entries 54307 to 54356), we obtained new data sets with phenotypic information for 191 combinations of amino acid changes in the RT and 571 in the PR. For each isolate, the available phenotypic data include a list of values representing the fold-increase of the IC_50 _for one or more antiretroviral drugs, obtained in comparison with a wild-type HIV clone.

Since the significance of each category might be different depending on the algorithm, we decided to compare the predictions by testing their ability to discriminate between susceptible and non-susceptible isolates. For most drugs, predictions given by DR_SEQAN were roughly similar to those obtained with other algorithms (Table 2). DR_SEQAN showed higher accuracy in the prediction of susceptibility to RT inhibitors, with figures above 75 % for all RT inhibitors, except stavudine. In contrast, our program was somewhat less efficient in the detection of isolates sensitive to several PR inhibitors, due to the conservative rules of the algorithm that often predicts partial resistance with only two secondary mutations. However, the percentage of drug-resistant isolates predicted to be susceptible to PR and nonnucleoside RT inhibitors was very low (estimated within the 4.6 – 16.3 % range) (Table 3). These type of errors were also relatively uncommon for nucleoside RT inhibitors (<20 %). The higher percentages obtained with various nucleoside RT inhibitors are a consequence of the lack of correlation between phenotypic data and clinical response observed with analogues whose resistance is mediated by the acquisition of significant ATP-dependent excision activity ([25], and references therein).

**Table 2:** Prediction accuracy of DR_SEQAN in comparison with other publicly available algorithms.

Inhibitor	Total no. of isolates	No. of drug-susceptible isolates^a^	Percentage of drug-susceptible isolates correctly predicted by the algorithms				
			DR_SEQAN	Retrogram	ANRS	HIVDB	RegaInst

Zidovudine (AZT)	135	48	79.2	64.6	72.9	68.8	72.9
Zalcitabine (ddC)	130	106	83.0	18.9	n.d.^b^	n.d.	24.5
Didanosine (ddI)	136	104	93.3	20.2	79.8	28.8	27.9
Lamivudine (3TC)	138	59	83.1	79.7	88.1	78.0	78.0
Stavudine (d4T)	136	85	60.0	38.8	41.2	37.6	67.1
Abacavir (ABC)	134	75	88.0	32.0	89.3	38.7	33.3
Nevirapine (NVP)	183	66	87.9	65.2	93.9	90.9	72.7
Delavirdine (DLV)	183	103	83.5	57.3	n.d.	82.5	58.3
Efavirenz (EFV)	180	97	79.4	48.5	67.0	64.9	49.5
Saquinavir (SQV)	548	278	65.8	73.4	89.9	73.4	80.9
Ritonavir (RTV)	510	237	67.9	75.1	84.0	88.6	77.2
Indinavir (IDV)	529	250	70.8	70.0	79.2	78.0	84.0
Nelfinavir (NFV)	567	224	66.1	70.5	80.4	73.7	77.7
Amprenavir (APV)	527	282	72.0	67.7	99.3	70.9	86.9
Lopinavir (LPV)	203	42 (99)	85.7 (43.4)	88.1 (60.6)	100 (87.9)	69.0 (29.3)	81.0 (45.5)

^a ^Drug susceptible isolates were defined as those that in comparison with the wild-type reference clone showed a fold-increase of the IC_50 _value for each drug, which was below 1.8-fold for AZT, 2.6-fold for ddC, 2-fold for ddI and d4T, 9-fold for 3TC, 4.5-fold for ABC, 6-fold for NVP, DLV and EFV, 1.7-fold for SQV and LPV, 2.5-fold for RTV and IDV, 3.6-fold for NFV and 2-fold for APV. Those cut-off values were consistent with those previously defined for the PhenoSense™ assay [24]. In the case of LPV, the values shown between parenthesis refer to the clinically relevant cut-off value of 10-fold [24].

^b ^n.d., not determined

**Table 3:** Analysis of discrepancies between phenotypic data and the predictions obtained with different interpretation algorithms.^a^

	Interpretation algorithm				
	
Inhibitor	DR_SEQAN	Retrogram	ANRS	HIVDB	RegaInst
Zidovudine (AZT)	2.8 (36)	0 (31)	0 (35)	0 (33)	2.8 (36)
Zalcitabine (ddC)	11.1 (99)	0 (20)	n.d.^b^	n.d.	1.4 (27)
Didanosine (ddI)	17.8 (118)	0 (21)	11.7 (94)	0 (30)	3.3 (30)
Lamivudine (3TC)	3.9 (51)	2.1 (48)	7.1 (56)	4.2 (48)	4.2 (48)
Stavudine (d4T)	12.1 (58)	2.9 (34)	0 (35)	0 (32)	0 (57)
Abacavir (ABC)	19.8 (81)	0 (24)	19 (84)	3.3 (30)	3.8 (26)
Nevirapine (NVP)	13.4 (67)	14.0 (50)	8.8 (68)	4.8 (63)	4.0 (50)
Delavirdine (DLV)	5.4 (93)	3.3 (61)	n.d.	3.4 (88)	3.2 (62)
Efavirenz (EFV)	11.5 (87)	12.8 (47)	10.8 (65)	1.6 (63)	6.3 (48)
Saquinavir (SQV)	8.9 (201)	8.1 (222)	30.0 (357)	6.4 (218)	14.4 (263)
Ritonavir (RTV)	4.6 (175)	5.4 (188)	6.5 (213)	4.1 (219)	7.6 (198)
Indinavir (IDV)	5.8 (190)	5.4 (185)	6.2 (211)	4.9 (205)	7.9 (228)
Nelfinavir (NFV)	5.6 (160)	6.0 (168)	13.9 (209)	2.9 (170)	13.0 (200)
Amprenavir (APV)	8.9 (223)	11.6 (216)	42.0 (483)	3.8 (208)	19.4 (304)
Lopinavir (LPV)	16.3 [*0*]^c ^(43)	54.8 [*28.6*] (84)	61.6 [*22.3*] (112)	3.2 [*3.2*] (31)	37.5 [*17.9*] (56)

^a ^The reported values are percentages of isolates that are predicted to be sensitive to the inhibitor (**S **category in Table 1), but show a fold-increase of the IC_50 _for the inhibitor, which is above the cut-off values given in Table 2. Numbers in parenthesis indicate the total number of isolates predicted to be susceptible to the drug in the analyzed data set.

^b ^n.d., not determined.

^c ^The numbers shown between brackets represent the percentages obtained using a 10-fold cut-off value.

The comparative analysis shows that DR_SEQAN provides coherent results with the other algorithms, while avoiding the large discrepancies between predictions and phenotypic data found with various drugs using other interpretation algorithms. Thus, discrepancies between the phenotypic data collected in the Stanford data set and the predictions given by DR_SEQAN occurred in only 16.3 % of the isolates predicted to be susceptible to lopinavir, while in the case of Retrogram, ANRS or RegaInst algorithms this figure increased up to >37 %, using a cut-off value of 1.7-fold (Table 3). Moreover, the higher accuracy of DR_SEQAN was also observed using a clinically relevant cut-off value of 10-fold. Reduced accuracies in lopinavir predictions could be attributed to inefficient rules that were established when the available information on lopinavir resistance was rather limited.

Other discrepancies between the predictions given by the different algorithms have been observed with zalcitabine, didanosine, stavudine and abacavir. For those RT inhibitors, the interpretation algorithms of DR_SEQAN and ANRS showed similar results, while displaying higher accuracy in the detection of isolates susceptible to nucleoside RT inhibitors (Table 2). In comparison with the other algorithms, thymidine analogue resistance mutations have a smaller impact on predictions carried out with DR_SEQAN or with the ANRS algorithm. However, mutational patterns conferring resistance to zalcitabine, didanosine, stavudine and abacavir are complex, and the contribution of thymidine analogue resistance mutations (M41L, D67N, K70R, L210W, T215F/Y, K219Q/E) is poorly-defined *in vivo*, particularly when other mutations such as L74V or M184V are present [4,15].

Although phenotypic data and predictions obtained with different interpretation methods give consistent results, it should be noted that phenotypic data do not always correlate well with the clinical response in infected patients [8,23,26], and a validation of currently used genotypic interpretation methods using clinical parameters (*i.e*. viral load, CD4 counts, etc.) would be necessary to make a more realistic assessment of the efficiency of DR_SEQAN in comparison with the other algorithms.

Together with the resistance interpretation, DR_SEQAN provides information on the effects of mutations on drug susceptibility. Thus, for each antiretroviral drug and combination of amino acid substitutions, the program displays the fold-increase of the IC_50 _relative to the wild-type reference virus, as determined in phenotypic assays. This value, which represents an average fold-increase of the IC_50 _when two or more papers report on the same combination of mutations, is obtained from the associated database. As in the prediction, drug resistance levels are highlighted in red, orange, yellow or green (Figure 1). High-level or significant resistance implies a >50-fold- or a >15-fold-increase of the IC_50 _(indicated in red or orange, respectively), relative to the value obtained with a reference HIV clone. For partial resistance (yellow), the increase of the IC_50 _should be higher than a specific threshold defined for each inhibitor: 6-fold for nevirapine; 5-fold for delavirdine and efavirenz; 4-fold for zidovudine, zalcitabine, didanosine, lamivudine and emtricitabine; 3-fold for abacavir and atazanavir; 2.5-fold for stavudine, indinavir, nelfinavir, amprenavir and lopinavir; and 2-fold for tenofovir, saquinavir, ritonavir and tipranavir. When the phenotypic data reveal that a combination of mutations does not confer resistance, the corresponding values are shown in green. On the other hand, a purple background is used to indicate that the mutation leads to a virus with increased susceptibility to the drug. Since IC_50 _values reported in the literature have been obtained using different phenotypic assays, the cut-off values used in the report have been arbitrarily chosen, and are different from those used in Tables 2 and 3, which correspond with specific cut-off values determined for the PhenoSense™ assay [24]. In addition to the fold-increases of the IC_50_, the user can obtain a list of bibliographic entries with the reported IC_50 _values and their corresponding links to PubMed by double-clicking on the corresponding cell.

## Conclusion

DR_SEQAN is an easy to use off-line application that provides expert advice on HIV genotypic resistance interpretation. As input data, the program uses HIV PR- and/or RT-coding sequences or a list of mutations. When the DNA sequence is provided, DR_SEQAN aligns the query sequence with a wild-type one, and identifies the mutations that are relevant for drug resistance. These mutations are used to run a genotypic interpretation algorithm that provides an estimate of the expected levels of resistance to all currently used antiretroviral drugs. The predictions given by DR_SEQAN are consistent with those provided by other algorithms and correlate well with phenotypic data. In addition, the program shows published phenotypic data for all possible combinations of mutations found in the query sequence. This information should be helpful to make an assessment on the robustness of the drug resistance prediction, based on available data collected from the literature.

## Availability and requirements

The entire DR_SEQAN software (including the drug susceptibility database) and documentation will be freely available for the scientific community from . Periodic information on algorithm updates and/or new releases of the drug susceptibility database will be given in our lab's web page at  or  (see research on Immunology and Virology). Currently, DR_SEQAN is available for PCs running Windows operating systems.

## Competing interests

The author(s) declare that they have no competing interests.

## Authors' contributions

CG developed and implemented the tool, tested the efficiency of the prediction algorithm and revised the manuscript. LM-A supervised the project and wrote the manuscript.

## Pre-publication history

The pre-publication history for this paper can be accessed here:



## Supplementary Material

Additional File 1 Genotypic interpretation algorithm (DR_SEQAN version 1.0). It contains the specific sets of rules used by the software to predict resistance to antiretroviral drugs. Click here for file
