# Supplementary material for: DR_SEQAN: a PC/Windows-based software to evaluate drug resistance using human immunodeficiency virus type 1 genotypes
Source: BMC Infect Dis. 2006 Mar 8;6:44. doi: 10.1186/1471-2334-6-44 (PMC1421411; doi:10.1186/1471-2334-6-44)
Supplement: Additional File 1 — Genotypic interpretation algorithm (DR_SEQAN version 1.0). It contains the specific sets of rules used by the software to predict resistance to antiretroviral drugs. [file 1471-2334-6-44-S1.pdf]

Genotypic interpretation algorithm (DR_SEQAN version 1.0)

1. Nucleos(t)ide analogue RT inhibitors

Drug	High-level resistance (**red**)	Significant resistance (**orange**)	Partial resistance (**yellow**)
Zidovudine (AZT)	**1.** (T69SSS, T69SSG or T69SSA) + T215Y + at least 2 of (M41L, A62V, K70R, L210W)<br>**2.** F77L + Q151M + (A62V, V75I or F116Y)<br>**3.** M41L + (T215F/Y) + at least 2 of (D67N, K70R, L210W, K219E/Q) **(a)**<br>**4.** In the presence of Q145L or Q145M	**1.** M41L + (T215F/Y) **(b)**<br>**2.** (T69SSS, T69SSG or T69SSA) + T215Y<br>**3.** F77L + Q151M	**1.** In the presence of T69SSS, T69SSG, T69SSA, Q151M, T215F or T215Y<br>**2.** Combinations of 2 or more mutations of (M41L, D67N, K70R, L210W, T215F/Y, K219E/Q)<br>**3.** Combinations of 2 or more mutations of (A62V, V75I, F77L, F116Y, Q151M)
Zalcitabine (ddC)	**1.** (T69SSS, T69SSG or T69SSA) + T215Y + at least 2 of (M41L, A62V, K70R, L210W)<br>**2.** F77L + Q151M + (A62V, V75I or F116Y)<br>**3.** In the presence of Q145M	**1.** (T69SSS, T69SSG or T69SSA) + T215Y<br>**2.** F77L + Q151M	**1.** In the presence of K65R, T69D, T69G, T69SSS, T69SSG, T69SSA, L74V or Q151M<br>**2.** Combinations of 2 or more mutations of (A62V, V75I, F77L, F116Y, Q151M)
Didanosine (ddI)	**1.** (T69SSS, T69SSG or T69SSA) + T215Y + at least 2 of (M41L, A62V, K70R, L210W)<br>**2.** F77L + Q151M + (A62V, V75I or F116Y)<br>**3.** In the presence of Q145L or Q145M	**1.** (T69SSS, T69SSG or T69SSA) + T215Y<br>**2.** F77L + Q151M	**1.** If K65R, T69G, T69SSS, T69SSG, T69SSA, L74V or Q151M are present<br>**2.** Combinations of 2 or more mutations of (A62V, V75I, F77L, F116Y, Q151M)
Lamivudine (3TC)	**1.** (T69SSS, T69SSG or T69SSA) + T215Y + at least 2 of (M41L, A62V, K70R, L210W)<br>**2.** In the presence of M184I, M184V, Q145L or Q145M	**1.** (T69SSS, T69SSG or T69SSA) + T215Y	**1.** When codon 67 is deleted<br>**2.** In the presence of K65R, T69SSS, T69SSG, T69SSA, K70E or P157S<br>**3.** (E44A/D) + V118I<br>**4.** Combinations of 2 or more mutations of (A62V, V75I, F77L, F116Y, Q151M)

Drug	High-level resistance (**red**)	Significant resistance (**orange**)	Partial resistance (**yellow**)
Stavudine (d4T)	**1.** (T69SSS, T69SSG or T69SSA) + T215Y + at least 2 of (M41L, A62V, K70R, L210W)<br>**2.** F77L + F116Y + Q151M + (K65R or V75I)<br>**3.** In the presence of Q145L or Q145M	**1.** (T69SSS, T69SSG or T69SSA) + T215Y<br>**2.** F77L + F116Y + Q151M	**1.** When codon 67 is deleted<br>**2.** In the presence of T69G, T69SSS, T69SSG, T69SSA, V75T, Q151M or T215Y<br>**3.** Combinations of 2 or more mutations of (A62V, V75I, F77L, F116Y, Q151M)<br>**4.** M41L + (T215F/Y)
Abacavir (ABC)	**1.** (T69SSS, T69SSG or T69SSA) + T215Y + at least 2 of (M41L, A62V, K70R, L210W)<br>**2.** F77L + Q151M + (A62V or V75I or F116Y)<br>**3.** M41L + D67N + L74V + (M184I/V) + L210W + (T215F/Y)<br>**4.** In the presence of Q145L or Q145M	**1.** M184V + 2 or 3 of (K65R, L74V, Y115F)<br>**2.** (T69SSS, T69SSG or T69SSA) + T215Y<br>**3.** F77L + Q151M	**1.** When codon 67 is deleted<br>**2.** In the presence of T69G, T69SSS, T69SSG, T69SSA or Q151M<br>**3.** Combinations of 2 or more mutations of (A62V, V75I, F77L, F116Y, Q151M)<br>**4.** M41L + (D67N or L210W) + (T215F/Y)
Emtricitabine (EMT)	**1.** In the presence of M184I or M184V	**1.** Combinations of 5 or more mutations of (M41L, D67N, K70R, L210W, T215F/Y, K219Q/E/H/N/R)	**1.** In the presence of K65R<br>**2.** D67N + K70R + (T215F/Y) + (K219E/N/Q)
Tenofovir (TDF)		**1.** (T69SSS, T69SSG or T69SSA) + T215Y + at least 2 of (M41L, A62V, K70R, L210W)<br>**2.** In the presence of K65R, Q145L or Q145M **(c)**	**1.** (T69SSS, T69SSG or T69SSA) + T215Y<br>**2.** Combinations of 3 or more mutations of (M41L, D67N, K70R, L210W, T215F/Y, K219E/Q)

(a) If an additional mutation of the group (K65R, L74V, W88G, E89K, L92I, A114S, S117T, S156A, Q161L, M164I, Y181C and M184V) is found, then the system warns that "Antagonistic mutations have been found in the sequence" (indicating the number of antagonistic mutations) (color code: **light red**). If M184V is found together with G333D/E, then the antagonistic effect is not expected and high-level resistance (color code: **red**) is predicted.

(b) If an additional mutation of the group (K65R, L74V, W88G, E89K, L92I, A114S, S117T, S156A, Q161L, M164I, Y181C and M184V) is found, then the system warns that "Antagonistic mutations have been found in the sequence" (indicating the number of antagonistic mutations) (color code: **light orange**). If M184V is found together with G333D/E, then the antagonistic effect is not expected and significant resistance (color code: **orange**) is predicted.

(c) The combination K65R + M184V is predicted as susceptible to tenofovir (color code: **green**).

2. Non-nucleoside RT inhibitors

Drug	High-level resistance (**red**)	Partial resistance (**yellow**)
Nevirapine (NVP)	In the presence of one mutation of (K103N, K103S, V106A, V106M, Q145L, Q145M, Y181C, Y181I, Y188C, Y188H, Y188L, G190A, G190C, G190E, G190Q, G190S, G190T, Y318W)	In the presence of one mutation of (A98G, L100I, K103H, K103T, V108I, V179D, F227C)
Delavirdine (DLV)	In the presence of one mutation of (L100I, K103H, K103N, K103T, V106A, Q145M, Y181C, Y181L, G190E, P236L) **(a)**	In the presence of one mutation of (K103S, V106M, E138A, V179D, Y188H, Y188L, M230L, Y318F)
Efavirenz (EFV)	In the presence of one mutation of (L100I, K103H, K103N, V106M, Q145L, Q145M, Y188L, G190C, G190Q, G190T, G190S)	In the presence of one mutation of (K101E, K103S, V106A, G190A, G190E, M230L)

(a) The system warns that "One antagonistic mutation has been found in the sequence" (color code: **light red**) when the following combinations are found in the HIV RT-coding region:
1.- (K103N, K103T or V106A) + (L100I, K103H, Q145M, Y181C, Y181L, G190E or P236L) + P225H, or
2.- V106A + (L100I, K103H, Q145M, Y181C, Y181L, G190E or P236L) + F227L
HIV isolates bearing the combinations: (1) (K103N, K103T or V106A) + P225H, or (2) V106A + F227L, are predicted to be susceptible to delavirdine (color code: **green**).

3. Protease inhibitors

Drug	Significant resistance (**orange**)	Partial resistance (**yellow**)
Saquinavir (SQV)	**1.** (G48V or I84A/V or L90M) + 1 or more mutations of (L10F/I/R/V, K20M/R, M36I/V, I54L/T/V, L63P, A71T/V, V82A/F/S/T)<br>**2.** 3 or more mutations of (L10F/I/R/V, K20M/R, M36I/V, G48V, I54L/T/V, L63P, A71T/V, V82A/F/S/T, I84A/V, L90M)	**1.** One mutation of (G48V, I84A/V, L90M)<br>**2.** Combinations of 2 mutations of (L10F/I/R/V, K20M/R, M36I/V, I54L/T/V, L63P, A71T/V, V82A/F/S/T), except those involving 2 mutations of the group (L10F/I/R/V, M36I/V, L63P)
Ritonavir (RTV)	**1.** (V82A/F or I84A/V) + 1 or more mutations of (R8Q, L10F/I/R/V, K20M/R, L24I, V32I, M46I/L, F53L, I54L/T/V, L63P, A71T/V, G73S, V77I, V82T, L90M)<br>**2.** 3 or more mutations of (R8Q, L10F/I/R/V, K20M/R, L24I, V32I, M46I/L, F53L, I54L/T/V, L63P, A71T/V, G73S, V77I, V82A/F/T, I84A/V, L90M)	**1.** One mutation of (V82A/F, I84A/V)<br>**2.** Combinations of 2 mutations of (R8Q, L10F/I/R/V, K20M/R, L24I, V32I, M46I/L, F53L, I54L/T/V, L63P, A71T/V, G73S, V77I, V82T, L90M), excluding the combination L63P + V77I
Indinavir (IDV)	**1.** (V82A/F or I84A/V or M46I/L) + 1 or more mutations of (L10F/I/R/V, K20M/R, L24I, I54L/T/V, L63P, A71T/V, G73S, V82T, L90M, I93L)<br>**2.** 3 or more mutations of (L10F/I/R/V, K20M/R, L24I, M46I/L, I54L/T/V, L63P, A71T/V, G73S, V82A/F/T, I84A/V, L90M, I93L)	**1.** One mutation of (M46I/L, V82A/F, I84A/V)<br>**2.** Combinations of 2 mutations of (L10F/I/R/V, K20M/R, L24I, I54L/T/V, L63P, A71T/V, G73S, V82T, L90M, I93L), except those involving 2 mutations of the group (L10F/I/R/V, L63P, A71T/V, I93L)
Nelfinavir (NFV)	**1.** In the presence of D30N or (N88D/S)<br>**2.** L90M + 1 or more mutations of (L10F/I/R/V, K20M/R, M36I/V, M46I/L, I54L/T/V, A71T/V, G73S, V77I, V82A/F/S/T, I84V, I93L)<br>**3.** 3 or more mutations of (L10F/I/R/V, K20M/R, D30N, M36I/V, M46I/L, I54L/T/V, A71T/V, G73S, V77I, V82A/F/S/T, I84V, N88D/S, L90M, I93L)	**1.** In the presence of L90M<br>**2.** Combinations of 2 mutations of (L10F/I/R/V, K20M/R, M36I/V, M46I/L, I54L/T/V, A71T/V, G73S, V77I, V82A/F/S/T, I84V)
Amprenavir (APV)	**1.** In the presence of I84V **(a)**<br>**2.** I50V + 1 or more mutations of (L10F/I/R/V, V32I, M46I/L, I54L/V, V82F/I, L90M) **(a)**<br>**3.** 2 or more mutations of (L10F/I/R/V, V32I, M46I/L, I54L/V, V82F/I, L90M) **(a)**<br>**4.** 3 or more mutations of (L10F/I/R/V, V32I, M46I/L, I50V, I54L/V, V82F/I, L90M) **(a)**	**1.** In the presence of one mutation of (V32I, I50V, I54L/V, V82F/I, L90M) **(b)**

Drug	Significant resistance (**orange**)	Partial resistance (**yellow**)
Lopinavir (LPV)	**1.** (I50V or I54A/M/S/T/V or V82A/F/S) + 5 or more mutations of (L10F/I, G16E, K20I/M, V32I, L33F, E34Q, K43T, M46I/L, I47A/V, G48M/V, I50V, I54A/M/S/T/V, Q58E, L63T, G73T, T74S, V82A/F/S, L89I/M)<br>**2.** 7 or more mutations of (L10F/I, G16E, K20I/M, V32I, L33F, E34Q, K43T, M46I/L, I47A/V, G48M/V, I50V, I54A/M/S/T/V, Q58E, L63T, G73T, T74S, V82A/F/S, L89I/M)	**1.** Combinations of 2 or more mutations of (L10F/I, G16E, K20I/M, V32I, L33F, E34Q, K43T, M46I/L, I47A/V, G48M/V, I50V, I54A/M/S/T/V, Q58E, L63T, G73T, T74S, V82A/F/S, I84A/C/V, L89I/M)
Atazanavir (ATZ)	**1.** Combinations of 5 or more mutations of (L10F/I/V, K20I/M/R, L24I, L33F/I/V, M36I/L/V, M46I/L, G48V, I50L, I54L/V, L63P, A71I/T/V, G73A/C/S/T, V82A/F/S/T, I84V, L90M)<br>**2.** Combinations of 4 or more mutations of (L10F/I/V, G16E, L33F/I/V, M46I/L, D60E, I84V, I85V, L90M)	**1.** Combinations of 3 or 4 mutations of (L10F/I/V, K20I/M/R, L24I, L33F/I/V, M36I/L/V, M46I/L, G48V, I50L, I54L/V, L63P, A71I/T/V, G73A/C/S/T, V82A/F/S/T, I84V, L90M)<br>**2.** Combinations of 3 mutations of (L10F/I/V, G16E, L33F/I/V, M46I/L, D60E, I84V, I85V, L90M)
Tipranavir (TPV)	**1.** V82T + I84V + 4 or more mutations of (L10F/I/R/V, K20I/M/R/T, L24I, D30N, V32I, L33F/I/V, M36I/L/V, M46I/L, I47V, G48M/V, I50V, I54A/L/M/S/T/V, A71I/T/V, G73A/C/S/T, V77I, N88D/S, L90M)<br>**2.** I84V + L90M + 4 or more mutations of (L10F/I/R/V, K20I/M/R/T, L24I, D30N, V32I, L33F/I/V, M36I/L/V, M46I/L, I47V, G48M/V, I50V, I54A/L/M/S/T/V, A71I/T/V, G73A/C/S/T, V77I, V82A/F/I/S/T, N88D/S)	**1.** Combinations of 2 or more mutations of (L33F, V82A/F/I/S/T, I84V, L90M)<br>**2.** Combinations of 12 or more mutations of the group (L10V, I13V, K20M/R/V, L33F, E35G, M36I, K43T, M46L, I47V, I54A/M/V, Q58E, H69K, T74P, V82L/T, N83D, I84V)

(a) The message indicating that "One or two antagonistic mutations have been found in the sequence" (color code: **light orange**) appears in isolates having the specified combination of mutations plus the amino acid substitutions K20T or N88S.
(b) HIV isolates with the combination (K20T or N88S) + (L10F/I/R/V, V32I, M46I/L, I50V, I54L/V, V82I/F or L90M), in their PR-coding region are predicted to be sensitive to amprenavir (color code: **green**).

<u>**Note**</u>: For each drug and combination of mutations, the program assigns the highest level of resistance compatible with the rules of the algorithm. For example, for zidovudine resistance predictions, the combination of mutations A62V + F77L + Q151M is compatible with high-level resistance (rule 2), significant resistance (rule 3) and partial resistance (rules 1 and 3). Therefore, DR_SEQAN's algorithm predicts high-level resistance to the drug following its hierarchical procedure.
